# Is the tobacco control movement misrepresenting the acute cardiovascular health effects of secondhand smoke exposure? An analysis of the scientific evidence and commentary on the implications for tobacco control and public health practice

**DOI:** 10.1186/1742-5573-4-12

**Published:** 2007-10-10

**Authors:** Michael Siegel

**Affiliations:** 1 Social and Behavioral Sciences Department, Boston University School of Public Health, Boston, MA 02118, USA

## Abstract

While chronic exposure to secondhand smoke has been well recognized as a cause of heart disease in nonsmokers, there has been recent speculation about the potential acute cardiovascular effects of transient exposure to secondhand smoke among nonsmokers; in particular, the possibility that such exposure could increase the risk of acute myocardial infarction even in an otherwise healthy nonsmoker. This paper reviews the claims being made by a number of anti-smoking and public health groups regarding the acute cardiovascular effects of secondhand smoke exposure among otherwise healthy adults, analyzes the validity of these claims based on a review of the scientific evidence, and discusses the implications of the findings for tobacco control and public health practice. Based on the analysis, it appears that a large number of anti-smoking organizations are making inaccurate claims that a single, acute, transient exposure to secondhand smoke can cause severe and even fatal cardiovascular events in healthy nonsmokers. The dissemination of inaccurate information by anti-smoking groups to the public in support of smoking bans is unfortunate because it may harm the tobacco control movement by undermining its credibility, reputation, and effectiveness. Disseminating inaccurate information also represents a violation of basic ethical principles that are a core value of public health practice that cannot and should not be sacrificed, even for a noble end such as protecting nonsmokers from secondhand smoke exposure. How the tobacco control movement responds to this crisis of credibility will go a long way towards determining the future effectiveness of the movement and its ability to continue to save lives and protect the public's health.

## Introduction

Secondhand smoke has been recognized as a cause of heart disease in nonsmokers [1,2]. In one of the most recent and comprehensive reviews of the scientific evidence, the Office of Environmental Health Hazard Assessment of the California Environmental Protection Agency estimated that chronic exposure to secondhand smoke causes a 20% to 50% increase in the risk of cardiovascular disease, resulting in between 23,000 and 70,000 deaths from heart disease in the United States each year [1].

While the cardiovascular effects of chronic exposure to secondhand smoke have been well studied and conclusively determined, there has been recent speculation about the potential acute cardiovascular effects of transient exposure to secondhand smoke among nonsmokers [3]; in particular, the possibility that such exposure could increase the risk of acute myocardial infarction even in an otherwise healthy nonsmoker.

This paper reviews the claims being made by a number of anti-smoking and public health groups regarding the acute cardiovascular effects of secondhand smoke exposure among otherwise healthy adults, analyzes the validity of these claims based on a review of the scientific evidence, and discusses the implications of the findings for tobacco control and public health practice.

## Analysis

### The 30-minute claim

In a January 27, 2006 press release, the Washington, D.C.-based anti-smoking organization Action on Smoking and Health (ASH) issued a press release calling on policy makers to adopt broad bans on public smoking, including bans in outdoor places such as public sidewalks [4]. ASH supported its call for such nearly complete outdoor smoking bans by claiming that even acute, transient exposures to small amounts of secondhand smoke cause fatal heart attacks among nonsmokers and that the risk of such a fatal heart attack is actually equivalent to that of a smoker:

"Even for people without such respiratory conditions, breathing drifting tobacco smoke for even brief periods can be deadly. For example, the Centers for Disease Controls [CDC] has warned that breathing drifting tobacco smoke for as little as 30 minutes (less than the time one might be exposed outdoors on a beach, sitting on a park bench, listening to a concert in a park, etc.) can raise a nonsmoker's risk of suffering a fatal heart attack to that of a smoker [5]."

The claim was documented with citation to two scientific articles, one published by authors at the Centers for Disease Control and Prevention (CDC) [3] and the other by Otsuka et al. [6].

This claim is of tremendous potential public health significance. If it is indeed true that acute exposure to drifting tobacco smoke for as little as 30 minutes can cause a fatal heart attack in an otherwise healthy nonsmoker (not an asthmatic) and that the risk of a fatal heart attack in a nonsmoker exposed to secondhand smoke for 30 minutes is elevated to that of a smoker, then this would seemingly provide strong justification for banning smoking completely in all public places, indoors and outside.

ASH has continued to use this scientific claim to promote smoking bans, writing to all members of the New Hampshire House of Representatives and urging them to support a ban on smoking in restaurants and bars because "Even for some people without respiratory conditions, breathing drifting tobacco smoke for even brief periods can be deadly. For example, the Centers for Disease Controls [CDC] has warned that breathing drifting tobacco smoke for as little as 30 minutes can raise a nonsmokers risk of suffering a fatal heart attack to that of a smoker [7]." ASH also used this claim in promoting a nearly complete ban on outdoor smoking in Calabasas, California [8]. ASH reiterated and reemphasized the claim in a February 24, 2006 press release [9].

ASH went so far as to warn city officials that they could be held liable for negligence and for damages if members of the public started dying of heart attacks due to brief exposures to secondhand smoke in city parks or other outdoors places:

"In cases where drifting tobacco smoke was present and a nonsmoker suffered a heart attack, asthmatic attack, or other similar problems, the municipality which owns and operates the beach, park, playground, etc. could be liable since it was on notice of the known health dangers but failed to take the 'reasonable' step of banning smoking as taken by many other outdoor areas [5]."

### Evaluation of the 30-minute claim

There is simply no evidence that exposure to secondhand smoke for 30 minutes represents a fatal myocardial infarction threat to nonsmokers who do not have existing severe coronary artery disease. The reason is quite simple. It takes more than 30 minutes to develop atherosclerosis significant enough to cause coronary artery stenosis so severe that it could cause a heart attack. There is also no evidence that acute exposure to secondhand smoke causes catastrophic arrhythmias, acute embolic events, or coronary artery spasm, such that it could induce a heart attack in an individual without existing coronary disease.

It also appears to be untrue that the CDC claimed that a 30-minute exposure to secondhand smoke "can raise a nonsmoker's risk of suffering a fatal heart attack to that of a smoker." What CDC did claim was that "even 30 minutes of exposure to a typical dose of secondhand smoke induces changes in arterial endothelial function in exposed non-smokers of a magnitude similar to those measured in active smokers [[3], p. 981]." This statement was based on findings from a study conducted by Otsuka et al., published in 2001 in the *Journal of the American Medical Association *(*JAMA) *[6].

The relevant study [6] experimentally exposed nonsmokers to secondhand smoke for 30 minutes and analyzed changes in endothelial function, as measured by coronary flow velocity reserve (CFVR). The authors reported that acute secondhand smoke exposure resulted in reductions in endothelial function that were approximately equivalent to those observed in active smokers [6].

It is important to note that endothelial dysfunction, especially if transient, is not a direct predictor of myocardial infarction risk. What endothelial dysfunction indicates is the early process of atherosclerosis. As the authors concluded: "The present findings suggest that reduction of CFVR after passive smoking may be caused by endothelial dysfunction of the coronary circulation, an early process of atherosclerosis, and that this change may be one reason why passive smoking is a risk factor for cardiac disease morbidity and mortality in nonsmokers [[6], p. 440]."

What this means is that acute exposure to secondhand smoke can result in endothelial dysfunction in nonsmokers that if prolonged and repeated over a long time, could eventually result in atherosclerosis and heart disease. This study provides a potential mechanism for the observed increase in heart disease risk among involuntary smokers. It provides biologic plausibility for a causal relationship between chronic exposure to secondhand smoke and heart disease. But it does not suggest that an otherwise healthy nonsmoker could suffer a heart attack as a result of a 30 minute exposure to secondhand smoke, and it certainly does not mean that a nonsmoker's risk of a heart attack approaches that of a smoker after 30 minutes of exposure to secondhand smoke.

Importantly, eating a single high-fat meal can also cause significant endothelial dysfunction [10]. Plotnick et al., writing in *JAMA*, reported that: "A single high-fat meal transiently reduces endothelial function for up to 4 hours in healthy, normocholesterolemic subjects, probably through the accumulation of triglyceride-rich lipoproteins [[10]- p. 1682]." It would not be accurate to interpret this study as indicating that a high-fat meal could cause a heart attack; instead, it does provide some plausibility for an association between a high-fat diet and the development of atherosclerosis. The same is true with the Otsuka study. It provides evidence that chronic exposure to secondhand smoke could promote atherosclerosis by inducing changes in endothelial function; however, one cannot extrapolate from that and claim that a single acute exposure increases the risk of a heart attack.

In fact, the Otsuka study found that a 30-minute exposure to secondhand smoke actually had no effect on basal coronary blood flow [6]. This transient exposure did not compromise the coronary circulation, damage the heart, or reduce blood flow to the heart muscle under actual real-life conditions. On the contrary, the study reported that: "Passive smoking exposure had no effect on basal coronary flow velocity in either group [[6]- p. 436]." In other words, this study documents that, at least in patients without severe coronary artery disease, a 30-minute exposure to secondhand smoke does not present a threat of reducing coronary blood flow. So the very study that ASH is relying upon to make its scientific claims actually refutes the very claims that it is making.

In light of the evidence, the extrapolation and resulting claims that ASH is making appear to be inaccurate.

In addition, it might be considered slightly irresponsible to try to intimidate city officials by suggesting to them that if they don't ban smoking in a beach, park, or playground, they are putting nonsmokers at risk of dropping dead of a heart attack. There is simply no evidence that acute exposure to secondhand smoke can cause a heart attack in a healthy nonsmoker, as one cannot develop coronary artery disease in 30 minutes.

### The 20-minute claim

The misrepresentation of the science of the acute cardiovascular effects of secondhand smoke extends beyond ASH. Another anti-smoking group – SmokeFreeOhio – went even further than ASH by claiming that just 20 minutes of exposure to secondhand smoke increases the risk of a heart attack. In a fact sheet posted on its web site, entitled "The Dangers of Secondhand Smoke," SmokeFreeOhio claimed that: "After twenty minutes of exposure to secondhand smoke, a nonsmoker's blood platelets become as sticky as a smoker's, reducing the ability of the heart to pump and putting a nonsmoker at an elevated risk of heart attack [11]."

In addition, SmokeFreeOhio claimed that "Only 30 minutes of secondhand smoke exposure can cause narrowing of blood vessels, restricting the flow of blood and contributing to hardening of the arteries [11]."

The group also claimed that "In that same 30 minutes, changes to your blood boost your risk of building up fat deposits that could lead to heart attacks and strokes [11]."

And it claimed that "After 120 minutes of exposure, your heart rate variability is reduced, increasing the chance of an irregular heart beat that can itself be fatal or trigger a heart attack [11]."

### Evaluation of the 20-minute claim

I will consider each of these four claims, in order.

1. "After twenty minutes of exposure to secondhand smoke, a nonsmoker's blood platelets become as sticky as a smoker's, reducing the ability of the heart to pump and putting a nonsmoker at an elevated risk of heart attack."

The fact sheet backs up this claim by citing a study which shows that brief exposure to secondhand smoke decreases platelet sensitivity to prostacyclin [12].

In the relevant study, Burghuber et al. examined the acute effect of secondhand smoke exposure on the sensitivity of platelets to prostacyclin [12]. Platelet aggregation has been shown to play a significant role in arterial thrombosis, one of the later stage events in the atherosclerotic process [12]. Prostacyclin plays an inhibitory role in helping to decrease platelet aggregation and decreased sensitivity to prostacyclin, as observed in patients with atherosclerosis [12,13], may therefore increase the proclivity of platelets to aggregate and thus promote atherosclerosis. In this study, acute secondhand smoke exposure was found to decrease platelet sensitivity to prostacyclin [12]. The authors conclude that acute secondhand smoke exposure increases platelet aggregation in nonsmokers and that this finding warrants further investigation to determine whether chronic exposure to secondhand smoke may therefore increase the risk of thromboembolic disease.

Thus, there is evidence that after 20 minutes of secondhand smoke exposure, platelet aggregation increases and that it may increase to the level of that seen in an active smoker. Thus, there is scientific support for a statement that a nonsmoker's platelets become as sticky as a smoker's after 20 minutes of secondhand smoke exposure.

However, one cannot extrapolate from a transient increase in platelet aggregation to a reduced ability of the heart to pump and an elevated risk of a heart attack. If one were exposed to secondhand smoke repeatedly for a long period of time, then the constant and prolonged effects of secondhand smoke on platelets, along with the effects on endothelial dysfunction, could initiate and maintain the process of atherosclerosis. But it cannot occur in just 20 minutes.

Moreover, even chronic exposure to secondhand smoke does not reduce the ability of the heart to pump. What reduces the ability of the heart to pump is injury to the cardiac muscle, such as occurs in a heart attack, with cardiomyopathy, with certain arrhythmias, with myocardial disease, with cardiac tamponade, or with ventricular hypertrophy or valvular disease. But secondhand smoke exposure does not reduce the ability of the heart muscle to pump. Chronic exposure could lead to a heart attack, and that could certainly reduce the heart's ability to pump. But the claim that only 20 minutes of secondhand smoke exposure can reduce the heart's ability to pump seems inaccurate.

It is important to point out that acute emotional stress also increases platelet aggregation [14]. But rather than suggesting that emotional stress in an otherwise healthy individual reduces the heart's ability to pump and places the individual at increased risk of suffering a heart attack, this research instead provides a potential mechanism for the observed relationship between chronic emotional stress and increased myocardial infarction risk [15].

2. "Only 30 minutes of secondhand smoke exposure can cause narrowing of blood vessels, restricting the flow of blood and contributing to hardening of the arteries."

The fact sheet backs up this claim by citing the Otsuka study [6]. But as pointed out above, that study demonstrated that 30 minutes of exposure to secondhand smoke causes endothelial dysfunction, not that it causes atherosclerosis and restriction of blood flow.

3. "In that same 30 minutes, changes to your blood boost your risk of building up fat deposits that could lead to heart attacks and strokes."

The fact sheet backs up this claim by citing a study which demonstrates that brief exposure to secondhand smoke causes changes in lipid metabolism [16].

Valkonen and Kuusi studied the acute effect of secondhand smoke on low density lipoprotein (LDL) metabolism [16]. They examined the effect of a 30-minute exposure on three factors related to atherogenesis: the antioxidant defense ability of serum, the extent of lipid peroxidation, and the accumulation of LDL cholesterol in cultured human macrophages. The oxidation of LDL cholesterol and its subsequent accumulation in macrophages is recognized as a critical event in the atherosclerotic process [16-18]. In this study, a brief secondhand smoke exposure was found to result in an acute decrease in serum ascorbic acid, decreased serum antioxidant defense, decreased ability of LDL to resist oxidation, increased lipid peroxidation, and an increased rate of uptake of LDL by cultured macrophages [16]. The authors conclude that an acute exposure to secondhand smoke alters LDL metabolism in a way that can promote atherosclerosis and that this finding provides a plausible biological mechanism for the observed effects of chronic secondhand smoke exposure on coronary heart disease.

Thus, while an acute exposure to secondhand smoke has been shown to induce changes in LDL metabolism that could lead to the development of fat deposits if these changes were sustained over time, making an extrapolation to claim that a 30-minute exposure will cause these deposits to form and increase the risk of heart attacks and strokes seems unwarranted.

4. "After 120 minutes of exposure, your heart rate variability is reduced, increasing the chance of an irregular heart beat that can itself be fatal or trigger a heart attack."

The fact sheet backs up this claim by citing a study of the effects of a two-hour exposure in a smoking area at Salt Lake City Airport on heart rate variability [19].

Pope et al. studied the acute effect of secondhand smoke exposure on heart rate variability, a measure of cardiac autonomic function [19]. They studied the effect of a two-hour experimental exposure to secondhand smoke. Reduced heart rate variability has been shown to predict an increased risk of cardiac vulnerability in patients with acute myocardial infarction, in part due to predisposition to fatal ventricular arrhythmias [19-23]. In this study, tobacco smoke exposure was found to significantly decrease heart rate variability, with a 12% decline in the standard deviation of normal-to-normal heart beat intervals [19]. The authors conclude that acute secondhand smoke exposure may cause impaired cardiac autonomic function and this may provide a biological mechanism for the observed increase in cardiovascular mortality among individuals chronically exposed to secondhand smoke.

While the first part of the SmokeFreeOhio statement is therefore accurate, as there is evidence that acute exposure to secondhand smoke does reduce heart rate variability, the second part of the statement is inadequately supported by scientific evidence. The observed short-term decrease in heart rate variability in this study did not translate into an increased risk of an arrhythmia that could be fatal or trigger a heart attack. The authors did not report the occurrence of any fatal arrhythmias or heart attacks in their study. If an acute decrease in heart rate variability did cause any appreciable risk of a fatal arrhythmia, it is difficult to imagine that this study could have been approved by an institutional review board.

It is important to point out that air pollution also decreases heart rate variability, in a very similar way to the findings observed due to secondhand smoke. There are at least three studies which have documented that particulate air pollution changes heart rate variability, just like secondhand smoke [24-26]; however, one wouldn't warn the public that exposure to air pollution may trigger a fatal or catastrophic arrhythmia.

In light of the evidence, the extrapolations and resulting claims that SmokeFreeOhio made appear to be inaccurate.

### Review of the scientific evidence on the acute cardiovascular effects of secondhand smoke

In order to review the current state of knowledge on the acute cardiovascular effects of secondhand smoke, I relied upon the two most recent and comprehensive reports on this subject, prepared by the Office of Health Hazard Assessment of the California Environmental Protection Agency [1], and the United States Surgeon General [2] and identified all the studies mentioned in these reports which are relevant to the evaluation of the potential impact on the cardiovascular system of brief exposure to secondhand smoke. This brief review represents a synthesis of the evidence from all of the identified studies and reflects the current state of the scientific evidence to the limits of the author's knowledge.

Studies of the acute cardiovascular effects of secondhand smoke exposure have documented adverse effects in six areas: (1) artery elasticity; (2) endothelial function; (3) platelet activation; and (4) cardiac autonomic function (heart rate variability); (5) lipid metabolism; and (6) exercise tolerance.

#### Artery elasticity

A five minute exposure to secondhand smoke reduced aortic elasticity by 21% in patients undergoing cardiac catheterization for angina [27]. Recovery was observed 15 minutes after discontinuation of exposure.

#### Endothelial function

Otsuka et al. experimentally exposed nonsmokers to secondhand smoke for 30 minutes and found that acute secondhand smoke exposure resulted in reductions in endothelial function of the coronary arteries, as measured by coronary flow velocity reserve, that were approximately equivalent to those observed in active smokers [6]. The study did not examine the duration of the effect on endothelial function following cessation of exposure.

Kato et al. [28] sought to determine whether the reduction in endothelium-dependent vasodilation that is observed in chronic passive smokers [29,30] requires chronic secondhand smoke exposure or whether it is an acute phenomenon. They studied brachial artery vasodilation in response to acetylcholine (which is an endothelium-dependent response) and to nitroprusside (which is not an endothelium-dependent response) before and after 15 minutes of exposure to secondhand smoke. There were no differences in the observed responses before and after the secondhand smoke exposure to acetylcholine and nitroprusside. This suggests that the endothelial impairment caused by brief exposure to secondhand smoke is reversible. The authors concluded that the observed endothelial impairment in passive smokers is due to chronic rather than acute secondhand exposure.

Although it is possible that the differences in the results of the Kato et al. [28] and Otsuka et al. [6] studies are attributable either to a difference in exposure time (15 versus 30 minutes) or to a different artery being studied (brachial versus coronary), the available evidence does not support a conclusion that brief exposure to secondhand smoke causes a permanent impairment of endothelial function that can lead to the development of atherosclerosis.

#### Platelet activation

Burghuber et al. found that a 20-minute exposure to secondhand smoke decreased platelet sensitivity to prostacyclin, thus increasing the tendency for platelet aggregation [12]. Sinzinger and Kefalides also found that an acute exposure to 15 minutes of secondhand smoke decreased platelet sensitivity to the anti-aggregatory effects of prostaglandins [31]. Schmid et al. reported that a one hour exposure to secondhand smoke in nonsmokers significantly increased levels of thromboxanes in the blood, which is an indicator of platelet activation [32]. The effects lasted for six hours after exposure.

#### Cardiac autonomic function

Pope et al. found that a two-hour exposure to secondhand smoke produced a 12% decline in the standard deviation of normal-to-normal heart beat intervals [19]. This effect on heart rate variability was reversed upon cessation of exposure.

#### Lipid metabolism

Valkonen and Kuusi found that a 30-minute exposure to secondhand smoke decreased serum ascorbic acid, decreased serum antioxidant defense, decreased ability of LDL to resist oxidation, increased lipid peroxidation, and increased the rate of uptake of LDL by cultured macrophages [16]. The effects on serum ascorbic acid lasted six hours. Moffatt et al. reported that after a six-hour exposure to secondhand smoke, high density lipoprotein (HDL) cholesterol levels decreased by 37% among nonsmokers [33]. This change required more than 24 hours to reverse.

#### Exercise tolerance

Although several studies have demonstrated a substantial decrease in exercise tolerance in response to acute exposure to secondhand smoke among patients with existing coronary artery disease [34-37], the effects observed on exercise tolerance among healthy adults have been small [38] or absent [37,39].

Taken as whole, these studies demonstrate that acute, transient exposure to secondhand smoke has a number of cardiovascular effects that, if repeated over time, may increase the risk of developing coronary artery disease by initiating and promoting the process of atherosclerosis and thrombosis. However, these effects were found to be reversible and there was no evidence of any clinically significant compromise of the coronary circulation in any of the patients in these studies. Moreover, these studies have actually documented that there was no impairment of basal coronary blood flow in subjects acutely exposed to secondhand smoke [6] and in at least one study, subjects undergoing exercise stress testing on a bicycle ergometer experienced no change in their exercise tolerance in response to acute secondhand smoke exposure [37].

To the best of my knowledge, there is no additional evidence, beyond the information presented in the above review, that a brief exposure to secondhand smoke can cause non-transitory cardiovascular effects. This represents the extent of the evidence upon which the claims of tobacco control groups are based.

In short, these studies provide strong biologic plausibility to support the observed association between chronic secondhand smoke exposure and heart disease; however, they do not provide evidence of any clinically meaningful effects of a single, acute, transient exposure.

In summary, there is simply no evidence from the existing data that a single, transient, acute exposure to secondhand smoke can or does increase the risk for myocardial infarction, nor is there any identified mechanism by which this could occur.

What one could conceivably argue is that for a patient with existing severe coronary artery disease, an acute exposure to secondhand smoke could cause an activation of platelets such that the final event in the process by which coronary stenosis leads to an acute myocardial infarction might be triggered. There is no direct evidence to suggest that this is the case, but I would not take issue with a group making an argument that people with severe coronary disease should avoid exposure to secondhand smoke because there is some possibility that in people who have coronary disease so severe that they are within one small physiologic change triggering a heart attack, exposure to secondhand smoke could potentially serve as the stimulus for that final physiologic instigating change. This, in fact, appears to be precisely what CDC has argued in its review of the issue of the reversibility and severity of the effects of transient, acute exposure to secondhand smoke [3].

If statements by public health groups were qualified by making it clear that the claims refer only to people with severe existing coronary artery disease, I would not necessarily question their validity. The problem is that claims being made by these groups are clearly suggesting to the public that for the general population, a single, short, transient exposure to secondhand smoke can trigger a myocardial infarction or other catastrophic or fatal cardiac event.

### Widespread dissemination of inaccurate scientific claims about the acute cardiovascular effects of secondhand smoke

The dissemination of these inaccurate claims about the acute cardiovascular effects of secondhand smoke exposure appears to go beyond simply the fact sheets produced by ASH and by SmokeFreeOhio. A physician who is also a tobacco control researcher and vice chairman of the Montana Tobacco Advisory Board reportedly told the press that: "If you go into a restaurant for a sandwich, if you go into a bar for a beer and you get exposed to a heavy amount of second-hand smoke, you're just as at risk for a heart attack as a smoker [40]." The newspaper article in which this statement was reported informed the public that "studies in the 1990s began pointing to heart attacks that were happening very rapidly from short-term exposure to second-hand smoke [40]."

A quarter-page advertisement in the *New York Times*, taken out by the New York City Department of Health on June 25, 2002, stated: "Just 30 minutes of exposure to secondhand smoke can greatly increase your risk of heart attack [41]."

A web site of another anti-smoking organization states that: "Twenty minutes of breathing secondhand smoke at levels similar to those measured in bars activates blood platelets involved in the clotting process as much as it does in pack-a-day smokers. These activated platelets increase the chances of getting a heart attack or stroke [42]". This section of the fact sheet is entitled "20 minutes exposure = smoking a pack a day." While the first sentence of this claim is accurate, and is based on the results of the Burghuber study [12], it seems somewhat of an inaccurate extrapolation to suggest that this single, transient episode of platelet activation puts one at risk of getting a heart attack or stroke, especially in an individual who does not have severe pre-existing coronary or vascular disease. It also seems misleading to suggest that the cardiovascular consequences of 20 minutes of secondhand smoke exposure is equivalent to that of smoking a pack of cigarettes per day.

The same fact sheet also claims that "30 minutes exposure = stiffened, clogged arteries" and that "Thirty minutes of secondhand smoke compromises a nonsmoker's coronary arteries to the same extent as in smokers [42]." While the Otsuka study [6] demonstrated that 30 minutes of secondhand smoke exposure can cause endothelial dysfunction, it did not provide any evidence that such an acute, transient exposure can caused "clogged arteries."

Another health organization made the claim that "Only 30 minutes of exposure can damage a non-smoker's heart and increases the risk of heart disease by 30% [43]." While epidemiologic studies do suggest that there is approximately a 30% increased risk of heart disease associated with chronic secondhand smoke exposure [1], this risk obviously cannot accrue in just 30 minutes.

An Americans for Nonsmokers' Rights fact sheet claims that "Even a half hour of secondhand smoke exposure causes heart damage similar to that of habitual smokers. Nonsmokers' heart arteries showed a reduced ability to dilate, diminishing the ability of the heart to get life-giving blood [44]."

Similar claims appear on the web sites of many other health and anti-smoking organizations [45-67] (see Appendix for the specific claims). Note that most of these communications claimed that 30 minutes of secondhand smoke exposure causes heart damage, narrowing of the coronary arteries, clogged coronary arteries, or reduced coronary blood flow, even though the Otsuka studied (which is cited for many of these claims) actually documented that there is no reduction in coronary blood flow in nonsmokers exposed to secondhand smoke for 30 minutes. It is clear that the inaccurate portrayal of the acute cardiovascular effects of secondhand smoke exposure on individuals without severe existing coronary disease is a widespread phenomenon.

### A tobacco control researcher responds

Although I am a strong advocate for smoke-free workplace policies, have testified in support of bar, restaurant, and workplace smoking bans in more than 50 cities, and have published a number of articles documenting the high secondhand smoke exposure and serious resulting health effects among bar and restaurant workers [68-72], I do not believe it is appropriate (or even necessary) to stretch the science and use inaccurate or misleading claims to support smoking bans. Because of my concern for the scientific integrity and long-term credibility of the tobacco control movement, I have publicly criticized tobacco control organizations for using inaccurate scientific claims to promote our policy goals [73-75].

In response to my questioning of the validity of these types of scientific claims being made by many anti-smoking groups, I have been personally attacked, publicly condemned, accused of being a traitor, accused of being funded by tobacco companies, called a fanatic, and have had my opinions censored by a prominent tobacco control policy discussion list-serve, from which I was expelled because advocates were apparently unhappy with my expressing dissent from the established dogma of the movement [76]. In my expulsion from the tobacco policy (tp-talk) discussion list-serve, I was told that the list-serve "made the dictatorial (but perhaps benevolent) decision to remove Mike Siegel from tp-talk today. I felt that his posts lately have interfered with the quality of the listserv messages. I suspect I'll be the subject of a blog posting about how he's been kicked off a tobacco control listserv, but I can deal with that" [76].

What is really being said is that I disagreed with some of the dogmatic views of the movement. In this case, that's what interference with the quality of the discussion means: disagreeing with the mentality of the movement. There is apparently no room for dissent in the tobacco control movement, and dissent is met not only with personal attacks, but with outright censorship.

Unfortunately, the one type of response I have not received is a scientific justification for the claims that are being made or a refutation of my scientific arguments. The general approach has been to attack ad hominem, rather than to directly confront the arguments being made. For this reason, I have come to the impression that the tobacco control movement does not allow room for any difference of opinion, and that those who dissent with any aspect of the prevailing wisdom must be discredited, attacked, and silenced. I sense a rather McCarthyistic element in the tobacco control movement. Whether the scientific arguments I have made are valid or not is up for question and debate; the unwillingness of the movement to entertain a discussion of the validity of its scientific claims, on the other hand, is a dangerous element in a public health movement.

This may be exactly the type of problem that Rothman predicted in his commentary which suggested that focusing on the scientist rather than on the merits of the science could lead to a "new McCarthyism in science [77]." Rothman argued that every piece of scientific work and criticism should be judged solely on its scientific merit, and that any attacks on these works should be science-based, not ad hominem attacks. My experience revealed that tobacco control advocates and groups are falling into this trap; the response to my critical pieces has consisted entirely of ad hominem attacks and has been devoid of any discussion of the scientific merits (or lack thereof) of my work.

### A sentinel event in the tobacco control movement

The discussion presented here about the scientific basis for claims about the acute cardiovascular effects of secondhand smoke exposure relates to more than just an issue of scientific interpretation. I view this as a sentinel event in the tobacco control movement. To me, this is the moment when it stopped being about the science.

For the past 20 years, I have never been in the uncomfortable position of having to refute the claims being made by colleagues and organizations in the movement of which I am a part. I have always found that there was a sincere concern for the accuracy of scientific claims and a high degree of care and concern given to scrutinizing claims before they were made publicly. However, in the past year or so, I believe that there has been a marked change in the movement. There seems to be much less of a concern for scientific accuracy and for the first time that I am aware, I believe that scientific integrity is being sacrificed for the sake of promoting our agenda.

I believe that the way in which the tobacco control movement responds to what I call the "30 minute" and "20 minute" claims is going to go a long way to determining not only the scientific integrity of the tobacco control movement, but also the reputation and credibility of the movement in the eyes of the media and the public for a long time to come.

The problem is that the media and the public cannot necessarily discern an accurate scientific claim from an inaccurate one. They cannot differentiate between a claim that is based on solid scientific evidence and one that represents a wild exaggeration or errant extrapolation from scientific data. Once the movement starts to make a number of widespread claims that are not scientifically supported, then the credibility of all of our public claims, even the legitimate ones, becomes suspect. And without credibility, the effectiveness of the movement in promoting its policy agenda will be greatly undermined.

But in addition to the potential consequences of the widespread dissemination of unsupported scientific claims for the credibility of the tobacco control movement, there is perhaps a more important consequence: the implications of this conduct in light of the ethical bases for the practice of tobacco control and public health.

### Implications for tobacco control and public health practice

It is important to recognize that the principle of providing accurate health information is a basic ethical principle of public health [78-81]. A recent editorial in the *American Journal of Public Health *emphasized "the responsibility of public health practitioners and scientists to conduct their practices ethically" [[78]- p. 1094]. Dickens suggests that the kind of ethical concerns that apply to individual research with human subjects should also apply at the collective level, and not only to research, but to the very practice of public health.

The provision of accurate health information to the public is recognized as a core ethical principle of public health practice. A recent article in *Tobacco Control *articulates the basis of the public's right to accurate health information from public health organizations [80]. Kozlowski and Edwards describe the public's right to accurate health information as deriving from the principles of autonomy and self-determination, and note that this right is supported by the Universal Declaration of Human Rights. According to Kozlowski and Edwards: "Individuals have a right to health relevant information; without it they cannot make meaningful health choices. Promoting and ensuring access to available knowledge is an obligation that follows from this right. Tobacco control information campaigns have sometimes fallen short of meeting the obligation of health relevant information. Failure can take many forms. Not informing that a product or activity involves health risks is one obvious example. Providing wrong or incomprehensible information would be another. Saying too little can also be deceptive and a violation of rights" [[80]- p. ii3].

The authors of this paper specifically address the issue of web-based health communications: "Much of the health communication we discuss employs the internet, and ethical guidelines have been established specifically for the internet (as is discussed in the US Healthy People guidelines in health communication and health literacy). These guidelines are unambiguous on honesty: 'Be truthful and not deceptive.' They emphasize the importance of providing accurate and well supported information. There is no allowance for the use of deception in web based health communications" [[80]- p. ii4].

Here, another important ethical concern deserves emphasis. It is not enough, according to these ethical guidelines, to simply provide information that is devoid of factual misrepresentations. Public communications must also be well supported by scientific evidence and should not be misleading, even if they are factually accurate.

## Conclusion

While there is ample evidence that chronic exposure to secondhand smoke increases the risk of cardiovascular disease, and therefore heart attack risk [1], and there is some suggestive evidence that acute exposure to secondhand smoke may present some degree of risk to individuals with existing severe coronary artery disease, there appears to be no scientific basis for claims that brief, acute, transient exposure to secondhand smoke increases heart attack risk in individuals without coronary disease, that it increases such risk to the level observed in smokers, that it can cause atherosclerosis, that it can cause fatal or catastrophic cardiac arrhythmias, or that it represents any other significant acute cardiovascular health hazard in nonsmokers.

In light of this, the claims that are being widely disseminated by a large number of tobacco control groups appear to be scientifically unjustified and inaccurate.

The dissemination of inaccurate information by anti-smoking groups to the public in support of smoking bans is unfortunate because it may harm the tobacco control movement by undermining its credibility, reputation, and effectiveness.

While anti-smoking groups may provide a utilitarian-based argument that these inaccurate and/or misleading communications are doing more good than harm in the long run because they are helping to promote smoke-free policies which will protect the public's health and save lives, the problem is that even if this were true, disseminating inaccurate information represents a violation of basic ethical principles that are a core value of public health practice that cannot and should not be sacrificed. The ends do not justify the means, especially when those means are violating principles of autonomy and self-determination that form the essential bases for free societies. These are values which cannot and should not be trodden upon by public health organizations simply to promote a favored policy.

How the tobacco control movement responds to this crisis of credibility will go a long way towards determining the future effectiveness of the movement and its ability to continue to save lives and protect the public's health.

## Abbreviations

ASH: Action on Smoking and Health

CDC: Centers for Disease Control and Prevention

CFVR: Coronary flow velocity reserve

JAMA: Journal of the American Medical Association

LDL: Low-density lipoprotein

## Competing interests

The author(s) declare that they have no competing interests.

## Appendix 1

### Examples of inaccurate scientific claims made by health and anti-smoking groups on acute cardiovascular effects of secondhand smoke exposure

#### Action on Smoking and Health

"Even for people without such respiratory conditions, breathing drifting tobacco smoke for even brief periods can be deadly. For example, the Centers for Disease Controls [CDC] has warned that breathing drifting tobacco smoke for as little as 30 minutes (less than the time one might be exposed outdoors on a beach, sitting on a park bench, listening to a concert in a park, etc.) can raise a nonsmoker's risk of suffering a fatal heart attack to that of a smoker [5]."

#### SmokeFreeOhio

"Only 30 minutes of secondhand smoke exposure can cause narrowing of blood vessels, restricting the flow of blood and contributing to hardening of the arteries. In that same 30 minutes, changes to your blood boost your risk of building up fat deposits that could lead to heart attacks and strokes. After 120 minutes of exposure, your heart rate variability is reduced, increasing the chance of an irregular heart beat that can itself be fatal or trigger a heart attack. After twenty minutes of exposure to secondhand smoke, a nonsmoker's blood platelets become as sticky as a smoker's, reducing the ability of the heart to pump and putting a nonsmoker at an elevated risk of heart attack [11]."

#### TobaccoScam

"30 minutes exposure = stiffened, clogged arteries [42]."

#### Heart Foundation South Africa

"Only 30 minutes of exposure can damage a non-smoker's heart and increases the risk of heart disease by 30% [43]."

#### Americans for Nonsmokers' Rights

"Even a half hour of secondhand smoke exposure causes heart damage similar to that of habitual smokers. Nonsmokers' heart arteries showed a reduced ability to dilate, diminishing the ability of the heart to get life-giving blood [44]."

### British Heart Foundation

"Just 30 minutes exposure to tobacco smoke can affect the cells lining the coronary arteries and this can contribute to the development of atheroma narrowing the coronary arteries and reducing blood flow to the heart [45]."

#### Coalition for a Tobacco-Free Hawaii

"Thirty minutes of secondhand smoke compromises a non-smoker's coronary arteries to the same extent as in smokers. All of these effects not only increase the long term risks of developing heart disease, but also increase the immediate risk of heart attack [46]."

#### Action on Smoking and Health (UK)

"Short term exposure to tobacco smoke also has a measurable effect on the heart in non-smokers. Just 30 minutes exposure is enough to reduce coronary blood flow [47]."

#### Campaign for Tobacco-Free Kids

"as little as 30 minutes of exposure to secondhand smoke can trigger harmful cardiovascular changes, such as increased blood clotting, that increase the risk of a heart attack [48]."

#### Clearing the Air Scotland

"30 minutes exposure to second hand smoke is sufficient to reduce coronary blood flow in otherwise healthy adults [49]."

#### DuPage County Health Department

"30 minutes exposure = stiffened, clogged arteries [50]."

#### Tobacco Public Policy Center

" A recent study completed by Japanese researchers concluded that just 30 minutes of exposure to secondhand smoke can lead to hardening of the arteries in nonsmokers [51]."

#### American Lung Association of Oregon

"As few as 30 minutes of secondhand smoke exposure can impair coronary circulation in a non-smoker [52]."

#### Tobacco-Free Coalition of Oregon

"The journal article points out that even 30 minutes of exposure to secondhand smoke increases blood platelet 'stickiness,' which can lead to blood clots. In addition, arteries narrow after exposure to secondhand smoke, so smaller clots cause more damage, and there is an increase in heart rhythm problems associated with heart attacks [53]."

### Health Sponsorship Council (New Zealand)

"After 30 minutes – arteries affected. Non-smokers usually have arteries that can dilate and boost blood flow to the heart more efficiently than a smoker's arteries. But exposure to second-hand smoke compromises that advantage after 30 minutes, to the same degree as for a pack-a-day smoker [54]."

#### University of North Carolina Department of Family Medicine

"30 minutes of exposure = stiffened, clogged arteries [55]."

#### Michigan Smoke-Free Dining Petition Drive

"A half hour of exposure to secondhand smoke dramatically increases a person's short-term risk of heart attack [56]."

#### Tobacco-Free Iowa (no longer an active link – this is the cached page)

"Nonsmokers exposed to secondhand smoke for just 30 minutes experience hardening of the arteries [57]."

#### Tobacco Technical Assistance Consortium (TTAC)

"Short exposure to secondhand smoke hardens arteries: According to Japanese researchers who presented at a recent American Heart Association meeting, as little as 30 minutes of exposure to secondhand smoke can cause the arteries of nonsmokers to harden [58]."

#### Maricopa County Department of Public Health

"Nonsmokers exposed to secondhand smoke for just 30 minutes experience hardening of the arteries [59]."

#### Citizens Against Unhealthy Smoke-Filled Environments

"Just 30 minutes exposure to secondhand smoke can compromise the cardiovascular system of nonsmokers by reducing blood flow to the heart [60]."

#### Smokefree Islington (UK)

"A study published in the Journal Of The American Medical Association found that just 30 minutes' exposure is enough to reduce coronary blood flow [61]."

#### New York City Department of Health and Mental Hygiene

"Just 30 minutes of exposure to second-hand smoke produces some of the same physical reactions that would occur from long-term smoking, and increases the risk of heart disease in non-smokers [62]."

#### Clean Air for Everyone (C.A.F.E.) Iowa

"Nonsmokers exposed to secondhand smoke for just 30 minutes experience hardening of the arteries [63]."

#### Washington State Department of Health

"Only 30 minutes of secondhand smoke exposure may cause heart damage similar to that of regular smokers. This exposure can reduce the ability of the arteries close to the heart to expand, which reduces the ability of the heart to receive life-giving blood [64]."

#### Campaign for a Healthy and Responsible Tennessee

"The Journal of the American Medical Association reports that just 30 minutes of exposure to secondhand smoke changes blood chemistry and increases the risk of heart disease in non-smokers [65]."

#### Smoke-free Bristol (UK)

"Short-term exposure to second-hand smoke has a measurable effect on the heart in non-smokers – 30 minutes exposure is enough to reduce blood flow to the heart muscle [66]."

#### Tobacco Free Coalitions of Clark County and Skamania County

"As little as 30 minutes of secondhand smoke can lead to hardening of the arteries in nonsmokers [67]."
